# Supporting early career health investigators in Kenya: A qualitative study of HIV/AIDS research capacity building

**DOI:** 10.11604/pamj.2015.20.192.5964

**Published:** 2015-03-02

**Authors:** Joseph Daniels, Ruth Nduati, James Kiarie, Carey Farquhar

**Affiliations:** 1 Department of Global Health, University of Washington, USA; 2 Department of Pediatrics and Child, Health University of Nairobi, Kenya; 3 Department of Obstetrics and Gynaecology, University of Nairobi, Kenya; 4 Department of Medicine, University of Washington, USA; 5 Department of Epidemiology, University of Washington, USA

**Keywords:** Research capacity building, HIV, Early career investigators

## Abstract

**Introduction:**

Strategies to transfer international health research training programs to sub-Saharan African institutions focus on developing cadres of local investigators who will lead such programs. Using a critical leadership theory framework, we conducted a qualitative study of one program to understand how collaborative training and research can support early career investigators in Kenya toward the program transfer goal.

**Methods:**

We used purposive sampling methods and a semi-structured protocol to conduct in-depth interviews with US (N = 5) and Kenyan (N = 5) independent investigators. Transcripts were coded using a two-step process, and then compared with each other to identify major themes.

**Results:**

A limited local research environment, funding needs and research career mentorship were identified as major influences on early career researchers. Institutional demands on Kenyan faculty to teach rather than complete research restricted investigators’ ability to develop research careers. This was coupled with lack of local funding to support research. Sustainable collaborations between Kenyan, US and other international investigators were perceived to mitigate these challenges and support early career investigators who would help build a robust local research environment for training.

**Conclusion:**

Mutually beneficial collaborations between Kenyan and US investigators developed during training mitigate these challenges and build a supportive research environment for training. In these collaborations, early career investigators learn how to navigate the complex international research environment to build local HIV research capacity. Shared and mutually beneficial resources within international research collaborations are required to support early career investigators and plans to transfer health research training to African institutions.

## Introduction

Strategies to transfer international health research training programs to Africaninstitutions follow a 25-year history of capacity buildingin sub-Saharan Africa by international and local organizations [1–9]. This transfer involves transitioning from international to local leadership of training programs. A key focus of these international training programs has been to develop and facilitate research career paths, but few career models exist in Africa due to limited local research resources and institutional research policies [3, 10–14]. Training program transfer is dependent on these career pathways because established local investigators teach and mentor future cadres of investigators as a measure of research capacity. One approach used by many programs harnesses both local and international resources to support early career investigators to develop future research leaders [3, 4, 10, 15, 16]. Despite the success of many of these programs, there have been few studies that identify and discuss these resources that support early career investigators.

One of the programs used to develop local research careers in sub-Saharan Africa is the AIDS International Training and Research Program (AITRP), which was designed as a response to the Institutes of Medicine Report, Confronting AIDS, published in 1986 [17]. The report described the international aspects of the epidemic, including its impact on foreign policy and research. It was decided that to address the epidemic, there was a need for international HIV/AIDS research collaborations between investigators in the US and low-resource countries. One approach to develop research collaborations was to provide HIV/AIDS research training to early career medical doctors and investigators from the US and low-income countries. Through this dual training approach, US and international investigators research developed HIV/AIDS research projects over time in partnership and built their individual research career pathways. The AITRP provided the international arm of the training for investigators from low-resource settings while providing short-term international research training to US investigators.

**AITRP description:** in 1988, there were 8 AITRPs funded as training grants by the National Institutes of Health (NIH) Fogarty International Center (FIC), and these were sustained for the next 25 years. Each individual program had different training priorities, and we focus on one of these first 8 programs. This study focuses on the University of Washington (UW) AITRP that was primarily designed to provide HIV/AIDS research training to Kenyan medical doctors and to non-medical trainees interested in an epidemiology or basic science research career (Socio-Behavioral Research, Biostatistics and Biomedical Research) at the University of Nairobi.

The UW AITRP was designed to train HIV researchers and conduct collaborative research at the College of Health Sciences at the University of Nairobi. There was limited research training in the medical curriculum at the University of Nairobi when AITRP began, and there has been some progress to build a research curriculum at the institutional level with some support from recent programs like the Medical Education Partnership Initiative, but research remains limited. A large component of the research capacity at the University of Nairobi includes international health research collaborations that have been sustained for nearly 35 years. The long-term goals of AITRP was to develop HIV research leaders at the University of Nairobi to develop and sustain long-term research collaborations to address the global AIDS epidemic, and then to support the development of HIV research leaders over several grant cycles who would eventually take-on this training program in Kenya (Table 1). We represent each grant cycle of this AIRTP in Table I to demonstrate how the program changed over time to accomplish these goals. Also, we show in Table II how many Kenyan individuals were trained through the program. In addition to Kenyan trainees, this program provided short-term and long-term research training to US infectious disease fellows who would work on collaborative research projects with Kenyan trainees. Since the program began in 1988, there have been 56 US trainees, and 13 of these continue to collaborate with Kenyan researchers. This training model provided a mutual benefit so that US and Kenyan investigators were trained together and learned how to research collaboratively. The training program was designed to increase the numbers of investigators in the Kenya and US who could then participate in international collaborations to address the Kenyan and global HIV/AIDS epidemic. As the program responded to Kenyan training and research needs over time, the research capacity building process was refined to support the continuous development of investigators who would then take on the responsibility of running AITRP at the local level while maintaining international research collaborations (Table 2).

**Table 1:** uw AITRP Design per Grant Cycle 1988-2013

Grant Cycle	Goals	Curriculum	Focus Areas
**1988-1992**	Multidisciplinary research training and collaboration to address HIV/AIDS	MS, MPH and Short-term training in Epidemiology, Clinical Research, and Laboratory methods	Program start-up and training
**1993-1997**	Interdisciplinary training and research collaboration to address HIV/AIDS	MS, MPH and Short-term training in Epidemiology, Clinical Research, and Laboratory methods	Increased focus on long-term training
**1998-2002**	Long-term research collaborations, training for research independence and institutional strengthening	MS, MPH, PhD and short-term training in Epidemiology, Biostatistics, Health Services and Behavioral Sciences	Encouraged US faculty to develop long-term research in Kenya to support early career researchers Institutional capacity building
**2003-2007**	Long-term research collaborations, training for research independence and institutional strengthening	MS, MPH, PhD degrees Short-term training Curriculum tracks in research fields	Advanced in-country early career research grants Research infrastructure building in-country
**2008-2013**	Produce competent investigators who will return home to continue collaborative HIV/AIDS research and training	MS, MPH, PhD and short training in Epidemiology, Biostatistics and basic sciences	Transition AITRP to Kenya

Data from UW AITRP grant applications to the National Institutes of Health

This data was collected and analyzed in the UW AITRP offices

**Table 2:** Number of Kenyan Trainees by Grant Cycle and Training Type

Grant Cycle	Short-Term Training (Up to 4 Months)	Long-Term Training (Incl. Degree Seeking)	Total Training (Grant Cycle)
**1988-1992**	9	12	21
**1993-1997**	18	8	26
**1998-2002**	26	21	47
**2003-2007**	17	18	35
**2008-2013**	9	14	23
**Total (Training Type)**	79	73	152

**Complex leadership theory in health research career development:** Kenyan health research leadership is complex, meaning it requires learning how to navigate international and local resources to develop individual careers and support expanding research capacity. We apply complexity leadership theory here as a framework to describe early career development [9, 18, 19]. Here, complexity leadership theory considers how individuals within a research network, to include trainees, early career researchers and independent investigators, interact less through authority; rather, individuals lend their expertise to mentor in research training and career development, which allows for adaptation and creativity in these areas. Similarly, we found these characteristics of complexity leadership among AITRP fellows when we studied their motivations for an HIV/AIDS research career, and this leadership approach is increasingly emphasized within health professional training globally [9, 20]. We showed that Kenyan researchers who were motivated to conduct HIV/AIDS research assembled together professional and internship research experiences from high school through their undergraduate training in order to supplement the limitations in their research training at educational institutions. The ability to incorporate additional training experiences as a supplement to traditional training reflected the complexity of Kenyan HIV research training and served as leadership models for others to follow in their own training. However, these career paths remain ambiguous since research is not fully institutionalized in Kenyan higher education, and there is limited understanding of the complexity that early career researchers must navigate now toward an independent research career.

We conducted a qualitative study of participants in the UW AITRP in order to understand what factors influenced early career investigator development of Kenyan AITRP trainees and how these influenced the transfer plans of this training program to the University of Nairobi, a public institution. First, we describe the methods used in this study. Next, we present two key findings to inform the relationship between early career investigators and the transfer of this training program to Kenyan leadership. Finally, we discuss complexity leadership for early career researchers and the importance of a strategy that includes shared resources as a means to support early career investigators and describe implications for such international training programs

## Methods

In order to understand factors that influenced early career investigator development to support a local AITRP in Kenya, we wanted to collect the perspectives of established, independent HIV/AIDS investigators who completed this AITRP or mentored Kenyan trainees in the program and remain affiliated with either the University of Washington or Nairobi. Therefore, we conducted a qualitative study with Kenyan and US independent investigators (N = 10) affiliated with the training program at the University of Washington. We used purposive sampling and selected Kenyan (N = 5) and US (N = 5)using two criteria [21]. The first criterion for participants was that they needed to be either a former AITRP trainee (N = 8)or faculty member (N = 2). If a participant was a former AITRP fellow, then they needed to have 10 years or more of experience training and mentoring others in HIV/AIDS research. And, we selected two faculty who were not former AITRP fellows because they had more than 10 years of long-term research collaborations in Kenya. The second criterion for participants was that they had ongoing research collaborations in one of the four HIV/AIDS research fields(Epidemiology, Biomedicine, Biostatistics and Socio-Behavioral). These criteria allowed us to gather current and long-term perspectives of established US and Kenyan investigators on early career investigators in Kenya.

All participants completed a human subjects approved oral consent process for this qualitative study that was conducted as part of the International AIDS Training and Research Program at the University of Washington and its affiliation with the University of Nairobi. Interviews were conducted using a semi-structured interview protocol asking questions about individual engagement in the program, early career investigators, and their perspectives on program implementation and the research capacity building process in Kenya. The interviews were conducted in the US and Kenya, 40-90 minutes in length, and audio-recorded and transcribed for analysis. The initial analysis used five large code categories about health research based on the literature presented. The code categories for health research were: teaching, experience, environment, careers, and knowledge transfer. These codes were refined during the analysis, which generated a set of sub-codes that were used in second review of the transcripts [21, 22]. After the transcripts were coded, we compared the interviews to identify major themes about Kenyan early career investigator pathways and training program transfer.

## Results

The findings speak to the experiences of researchers at Kenyan national universities since participants completed training or did research at the University of Nairobi prior to AITRP. There are two major findings that speak to the factors that influenced early career research development and AITRP program transfer.

**Kenyan research environment and funding:** participants stated that training was not enough to ensure sustainable research careers. Based on their own experiences and their trainees, participants stated that the transition from trainee to early career and then to independent investigator takes time. Participants also stated that more senior investigators were needed to provide mentoring for early career investigators. Specifically, participants explained that the inability to support early career investigators stemmed from a limited research culture in Kenyan:

“Definitely it [research environment] is still a lot to be desired…. A lecturer doing something beyond coming to class is almost non-existent. They are interested in lecturing but not interested in medical research…” –Kenyan Investigator One.

“You have to hit a critical mass; a discomfort around happiness or desire to change, or the number of people who are willing to move change and then make things happen. It tips over. And, I think, it's the same thing with even this culture of research. We are perhaps getting to the point where are we are starting to gather critical mass for those who have been trained and are interested in re-thinking the research idea...” —Kenyan Investigator Two

These two AITRP investigators explained that there was a limited research culture and that university policies did not support research capacity building in Kenya. There were few investigators interested in research and able to mentor early career investigators. Also, participants stated that increasing the priority of research within universities is a long-term process of change. As the second investigator stated, a “tipping point” would need to occur to institutionalize locally research activities. To reach this tipping point requires that an increased number of independent investigator shave funding and can show a feasible career path for trainees and early career investigators. Also, the fact that there was limited local research funding continued to be a focus of the participants. At the time of this study, the concern was that not enough investigator shad been trained through this program to be independent investigators. Participants perceived that the individual investigator capacity in Kenya was still in transition and that there was need for more local investigator research funding for research, training and mentoring. As one medical investigator stated that captured the common perspective among the participants:

“I think right now, as it is set up here, we need to bring in more funding. The more you get, the more you get. And, from there, you can open up to all those questions like is it [local AITRP] a master's or PhD training program? Because if you start a PhD program, and people finish their PhDs and then they start running away from you to look for positions. So, you need to make the ground fertile. You need to establish a strong foundation to build on. If your foundation is not there and your building is high, it's just gonna wobble.” –Kenyan Program Investigator Three.

The concern of this medical investigator was that the funding in Kenya among investigators was not at a level to support degree programs and post-doctoral training. She elaborated on this funding need to explain that there were few independent investigators to financially support, train and mentor trainees and early career investigators. The participant argued that, without a solid financial foundation, those with advanced research training at the PhD level would leave for a supportive research environment. Specifically, “fertile ground” is more than funding for that participant; it includes a research environment that supports knowledge sharing to develop research collaborations and broad institutional support for investigator doing research. Fertile ground also meant increased research funding awarded to Kenyans so that those trained through AIRTP can mentor and support additional early career investigators. Participants described an emerging research career pathway in Kenya that continues to be negatively influenced by highly trained people leaving Kenya due to limited local research funding, insufficient mentoring and lack of policies favoring researchers and research activities.

Participants perceived that there was a gap in support between training and independent investigator status. This gap limited the ability of early career investigators, such as post-doctoral trainees, to develop their research career. As a result, participants perceived that early career investigators were more likely to leave Kenya for research opportunities elsewhere, which contributed to brain drain. Yet, participants stated that international collaborations could provide that “fertile ground”during the transition as the Kenyan research environment continued to develop support for local investigators and research training in institutions.

**International research and training engagements:** Kenyans who reached independent investigator status had access to additional research funding, yet the ability for early career investigators to gain access to their own funding was limited without US collaborators. One US participant explained that the process of applying for funding is challenging for new international investigators who often need to rely on US collaborators’ research projects for support:

“There is a sense of isolation from the system [international research community], and one must rely on the [AITRP] and its [other] funding. If you didn't have that connection, you would just float along and really not know how to begin to apply. ... [N]etworking is hard for me, and I imagine it is bewildering for these guys. There needs to be a long-term plan that is supportive with a budget to give them a chance to keep going.” –US Investigator 1

That US investigator explained that it is easy to become isolated in Kenya without international research networks, especially when it comes to funding research. Networking is challenging for scientists around the world, as the investigator states, and this research is particularly challenging for investigators in resource-limited settings. This investigator member elaborated later in this interview that networking involves creating linkages with groups of investigators conducting complementary and supportive work in order to access research opportunities. This networking begins during training in this AITRP and develops over time as both US and Kenyan investigators complete their training together and start their careers. Participants perceived that there are not many networks to access, but the ongoing engagement of US investigators and trainees in Kenya allowed for both increased and stable research networks for early career investigators who completed the training program. Specifically, participants elaborated on the role of US collaborators involved with AITRP. The most successful US investigators that supported and mentored a Kenyan investigator into independent status had sustained grant support with collaborating Kenyan institutions over the long-term. Grant funding supporting research activities was essential to mentoring trainees into the international research model and research independence for both Kenyan and US trainees and early career investigators. In the biomedical research arm of this AITRP, two US investigators had participated in the training program for 10 and 23 years, respectively. One of these investigators explained that her experience in the training program was that “it takes 10 years of sustained international research, training, and mentoring in order to develop one person in the field.” The other elaborated on the role of US collaborators once trainees returned home:

“The international collaborative nature of work [research] keeps people on budget salary. Trying to get a position at [an African institution] is hard. [One trainee] had to volunteer teach up to two years and then he finally got a position in a department…. We provide [funding support] here so they can start applying for grants to support themselves and [stay in research].” –US Investigator 2

This US investigator member explained that the transition from trainee to investigator, especially during the first two years, is daunting because there are limited opportunities to merge research with teaching. Further, it is difficult for trainees to find permanent positions within Kenyan public higher education institutions because of the limited resources to fund new investigators, and these positions are usually for teaching only and do not include research responsibilities. As a result, trainees are developing new career pathways that don't exist in their respective institutions and that bridge both research and teaching in their home countries. This transition is dependent on continued support of US investigator and their engagement in this training program. Thus, it was perceived that it would be highly unlikely, that without ongoing US investigator support engaging former trainees in international research, retention of early and senior investigators would be difficult given the uncertain levels of research funding and limited institutional support of faculty research at Kenyan public higher education institutions.

## Discussion

This qualitative study of one AIDS International Training and Research Program (AITRP) demonstrated that there are three major factors that influenced early career investigators in Kenya, which informed how participants envisioned the transfer of AITRP to the University of Nairobi. Research careers in Kenya compete with teaching responsibilities. The balance between teaching and research is due in part to a limited local research environment and lack of funding for such. As a result, this limited research environment and funding challenges were factors that negatively influenced early career investigators. Yet, Kenyan investigators and US collaborators mitigated these factors by providing mentorship and transitional funding for research. This highlights the effectiveness of sharing international and local resources to develop international training programs that will eventually be transferred to sub-Saharan African institution while maintaining and enhancing international collaborations for education and research. Not only do these collaborations contribute to research capacity building, these collaborations are mutually beneficial by training cadres of US HIV/AIDS investigators through research opportunities internationally in countries like Kenya.

**Applying complexity leadership theory to Kenyan HIV careers:** we applied complexity leadership theory to health research career development in Kenya to understand the factors that influenced this development [9, 18, 19]. We found that early career Kenyan researchers and their mentors navigated complex systems of international HIV research, and they had to be adaptive to sustain their careers. International and Kenyan independent investigators navigate training and mentoring early career researchers within an evolving Kenyan research environment that has financial and policy limitations. This training and mentoring works across institutional policies and practices in different countries and is a practice of negotiating these to support career pathway development over long periods of time. Early career researchers navigate ambiguity in their research careers that allows for flexibility and creativity especially when they are linked to an international research network. Navigating mentoring from both international and Kenyan investigators is necessary to learn how to adapt international and local resources to clinical studies locally. The ability of an early career researcher to secure complex leadership skills of managing multiple mentors, career ambiguity and creativity with research resources demonstrates leadership to others who want to pursue this career pathway. Ultimately, the early career research pathway must be adaptive to changing resources, like research funding priorities, while enhancing and maintaining local and international research networks for career development.

**Shared resources sustain early careers:** therefore, this study outlined two shared resources to support early career researchers and develop them as AITRP training and research leaders in Kenya. These were: 1) Human resources, and 2) Funding. The human resources were the US and Kenyan investigators interviewed and most were part of the AITRP training. This mutually beneficial training model developed US and Kenyan collaborations during training that evolved into research projects afterward. These established investigators served as mentors for new early career investigators while linking them to funding through research grants. Research career path development is an essential part of capacity building with in institutions without policies or financial resources to support investigator who do research [5, 11]. In the absence of these local resources, participants believed that former trainees should maintain the international connections they gained through training. This would position early career investigators within on-going international research collaborations where they could be co-mentored by US and Kenyan investigators as a means to support (financial, research design mentoring, grants manship etc.) their professional development and advance their research careers. Thus, this study demonstrates the efficacy of capacity building models that harness international and local resources to support research careers [4, 10, 15, 16].

**Early career researchers in AITRP transfer plans:** the transfer of a training program from international to local leadership is also dependent on human resources and funding to sustain research investigators at different stages in their careers[3, 16]. Early career investigators were seen as an important asset for the transfer of AITRP to Kenya. Specifically, the feasibility of this program transfer was perceived to be dependent on established independent investigators in Kenyan who could serve as mentors and investigators for a locally led AITRP. At this time, participants interpreted their independent investigator capacity as limited, and participants were concerned that brain drain would remain a reality, especially for those who had not established their research careers. They identified international and local resources that should be directed to the pool of early career investigators to address this limited capacity and solidify the research career pathway. Over time, this strategy of sharing international and local resources would increase the numbers of independent investigators in Kenya who could mentor and train in an AITRP locally. Complementary to supporting Kenyan early career researchers, continued support of US early career researchers would sustain the international collaborations developed during training and build these collaborations further through long-term research.

**Limitations:** there are some limitations of this study. We interviewed only independent investigators based at the Universities of Washington and Nairobi. This study could have benefited from interviews with early career investigators, Kenyan institutional leadership, and participants in other international training programs linked with the University of Nairobi. However, we were interested in understanding the perspectives of independent investigators affiliated with this AITRP who could discuss the current challenges and opportunities for early career investigators who receive AITRP support as it relates to the transfer of this program to Kenya. Based on our findings, we believe that additional research is needed with other international training programs to understand the development of early career researchers in Africa. Also, we focused only on public institutions here. We acknowledge that early career researchers at private and parastatal institutions in Kenya may have different career track experiences that deserve study.

## Conclusion

This study of AITRP offers three implications for training programs and international research collaborations that operate within public higher education institutions Kenya. The first implication is that the transfer of AITRP should continue the focus on mutual benefits of training future US and Kenyan investigators as collaborators, which can lead to collaborative research later in their careers. The second implication is that programs like AITRP, whether these are international or local, must be designed to support early career investigators both in the Kenya and the US as a mutually beneficial practice. This support may need to include expanded research funding dedicated to early career researchers. The third implication is that shared international and local resources are necessary to support early career investigators who will eventually play a critical role in building local research capacity and sustaining local research training programs.
